# Exploring Distress and Occupational Participation Among Older Canadians During the COVID-19 Pandemic

**DOI:** 10.1177/00084174231165832

**Published:** 2023-05-15

**Authors:** Elisabeth Vesnaver, Nicholas Dietrich, Renata Kirkwood, Jinhui Ma, Rhianna Guennel, Marla Beauchamp, Heather Keller, Luciana Macedo, Janie Astephan Wilson, Brenda Vrkljan

**Keywords:** Mental health, activities of daily living, regression analysis, qualitative research, Activités de la vie quotidienne, analyse de régression, recherche qualitative, santé mentale

## Abstract

**Background.** The coronavirus disease 2019 (COVID-19) pandemic disrupted daily life with corresponding implications on levels of distress. **Purpose**. To describe factors associated with high distress among community-dwelling older adults during the first lockdown and explore how occupational participation was managed. **Methods**. A mixed methods design whereby multivariate regression analysis of a survey (*N *= 263) identified factors associated with high distress, as per the Impact of Events of Scale-Revised (IES-R). Follow-up interviews with a sub-sample of those surveyed who reflected a range of IES-R scores were conducted (*N *= 32). **Findings**. Those with lower resilience and anxiety/depression had 6.84 and 4.09 greater odds respectively of high distress. From the interviews, the main theme, “Lost and Found,” and subthemes (Interruption and Disruption; Surving, not Thriving; Moving Forward, Finding Meaning) highlighted the process and corresponding stages, including adaptive strategies, by which participants navigated changes in their occupational participation. **Implications.** While the results suggest that many older adults, including those with high distress, were able to manage daily life under lockdown, some experienced ongoing challenges in doing so. Future studies should focus on those who experienced or who are at-higher risk for such challenges to identify supports that mitigate adverse consequences if another event of this magnitude occurs again.

## Résumé

**Description.** En perturbant le quotidien, la pandémie de COVID-19 a eu des répercussions sur les niveaux de détresse. **But.** Décrire les facteurs associés à une détresse élevée chez les personnes âgées vivant dans la collectivité durant le premier confinement, et explorer comment la participation occupationnelle a été gérée. **Méthodologie.** Une recherche à méthode mixte dans laquelle l’analyse de régression multivariée d’une enquête (*N* = 263) a permis d’identifier les facteurs associés à une grande détresse, selon le questionnaire *Impact of Events of Scale-Revised* (IES-R). Des entretiens de suivi ont été menés auprès d’un sous-échantillon de personnes interrogées ayant obtenu différents scores à l’IES-R (*N* = 32). **Résultats.** Les personnes moins résilientes et les personnes anxieuses ou dépressives étaient respectivement 6,84 et 4,09 fois plus susceptibles de souffrir d’une grande détresse. Les entretiens ont permis de dégager le thème principal, « Perdu/trouvé », et les sous-thèmes (Interruption et perturbation; En mode survie; Aller de l’avant, Trouver un sens), qui mettent en relief le processus et les étapes correspondantes, y compris les stratégies d’adaptation, par lesquels les participants ont fait face aux changements dans leur participation occupationnelle. **Conséquences**. Bien que les résultats suggèrent que de nombreuses personnes âgées, y compris celles ayant un niveau de détresse élevé, ont été capables de gérer leur vie quotidienne pendant le confinement, certaines ont eu des difficultés constantes à le faire. Les études futures devraient se concentrer sur les personnes qui ont connu ou qui sont plus susceptibles de connaître de telles difficultés, afin d’identifier des mesures de soutien pour atténuer les conséquences négatives si un autre événement de cette ampleur se produit.

## Introduction

When the global pandemic emergency was declared in mid-March 2020, there was a sudden and immediate change to daily life, as governments in Canada and other countries implemented public health measures to mitigate the spread of severe acute respiratory syndrome coronavirus 2 or coronavirus disease 2019 (COVID-19). These measures included isolation, quarantining, physical distancing, masking, and lockdowns (Public Health Agency of Canada (PHAC), 2022a). In the province of Ontario, the first lockdown lasted 4 months (Canadian Institute for Health Information, 2022). During this time, there was a heightened sense of distress, particularly among those aged 70 and older who have the highest rates of mortality from the virus (PHAC, 2022b). With vaccines not yet available, many community-dwelling older Canadians adhered to public health precautions thereby isolating themselves from family and friends.

Recent analysis from the Canadian Longitudinal Study on Aging involving 28,559 Canadians aged 44 and older examined the prevalence of depressive symptoms before the pandemic to the time period during the first lockdown (Raina et al., 2021). Compared to individuals aged 75 and older, they found that individuals aged 65−74 followed by those aged 55−64, and < age 55 had 1.15, 1.50, and 1.79 the odds of experiencing depressive symptoms after adjusting for socioeconomic status, loneliness, and number of chronic conditions respectively. While these findings suggest those in the oldest age group fared slightly better during the first lockdown, the authors expressed caution with this interpretation, as a significant portion of older adults had higher rates of depressive symptoms, as compared to their prepandemic levels. The relationship between age and depression persisted regardless of their health and/or social status. Hence, the question remains as to why some older people experienced such symptoms more than others. To address such a question, a more detailed study of their experiences is warranted.

Recent qualitative investigations undertaken with community-dwelling older adults in Canada (Rotenberg et al., 2021) and Sweden (Carlsson et al., 2022) suggest key differences in their experiences with navigating daily activities during the early months of the pandemic. In their interviews with older Canadians (aged 65–80) where the focus was on how COVID-19 public health measures were interpreted and applied to daily life among those with subjective cognitive decline, Rotenberg et al. (2021) described heightened feelings of fear, distress, and boredom in this population, as activities shifted online and became more sedentary. In Sweden, Carlsson and colleagues, using the four dimensions of occupation, doing, being, becoming, and belonging, noted that even in the face of extensive changes in routines and habits due to the pandemic restrictions, the need for engagement remained strong. While both studies alluded to the psychological impact of the pandemic on occupational participation, neither analysis included measures of mental health, including distress. In fact, Rotenberg et al. excluded those from their study who had scores indicative of depression (i.e., score ≤9 on Patient Health Questionnaire [Kroenke, Spitzer, &Williams, 2001])

Occupational participation, defined as “having access to, initiating and sustaining engagement in valued occupations within meaningful relationships and contexts” encompasses what individuals need to do, want to do, or are expected to do, which can differ depending on one's life stage, culture, and other circumstances (Egan & Restall, 2022, p. 76). Withdrawal, disruption, and changes in one's ability to participate in valued occupations in times of adversity, whether due to a life-threatening diagnosis or otherwise, have been linked to how individuals perceive their own identity and health (Vrkljan & Polgar, 2001). A systematic review conducted by a member of the authorship team that examined major events or life transitions in older adulthood, such as bereavement or retiring from paid employment, identified how the resulting distress from such an event, among other issues, can require formal intervention (Vrkljan et al., 2019).

Psychological distress has been characterized as one's emotional state, including anxiety and depression, that can arise in the face of external stressors where individuals differ in their respective vulnerability to such stressors (Matthews, 2016). Posttraumatic stress disorder (PTSD) refers to a medical diagnosis associated with a set of symptoms associated with an upsetting traumatic event, which can have long-lasting negative consequences on physical and mental well-being (American Psychiatric Association, 2022). After a traumatic event, like the recent pandemic, older adults are considered a high-risk group for PTSD due to existing health issues and other challenges, which can lead to further deterioration in function (Cook et al., 2017).

In spring 2020, our research team undertook a longitudinal survey to examine the impact of the COVID-19 pandemic on the mobility and participation of a group of community-dwelling older Canadians who were randomly recruited (see Beauchamp et al., 2021). Initial analysis of the baseline survey indicated a high prevalence of clinically significant levels of psychological distress, and, as such, was a covariate in a subsequent study involving this sample (Saunders et al., 2022). With this finding in mind, it was determined that to further understand how older adults managed their occupational participation early in the pandemic, exploring the level of distress experienced in this population and its predictors was necessary. Hence, the purpose of the current study was to describe factors associated with high distress among community-dwelling older adults during the first lockdown and to explore how those with a range of distress managed occupational participation during this time.

## Methods

### Study Design

This study takes a pragmatic approach and is rooted in the goal of producing clinically and socially useful research (Feilzer, 2010). We used a partially mixed methods exploratory design (quan→QUAL). Secondary analyses from our baseline survey (Beauchamp et al., 2021) identified predictors of those with different levels of distress (quan) and enabled orientation to a phenomenon that was explored in a subsequent dominant qualitative phenomenological interview study (QUAL) (Mayoh & Onwuegbuzie, 2013). Level of distress and corresponding predictors informed the selection of those interviewed where their respective experiences with managing occupational participation under lockdown were explored (Collins et al., 2006). Integration occurred at both the methods and interpretive levels. The qualitative sampling frame built upon the quantitative data and findings. Quantitative data informed the qualitative analysis. Finally, we integrated the qualitative and quantitative phases through narrative in the interpretation of the findings (Fetters et al., 2013). Interviews occurred 6–12 months after the baseline survey. Ethics for the survey and interviews were approved by the Hamilton Integrated Research Ethics Board of McMaster University (2020-10814-GRA).

### Data Collection

#### Survey Questionnaire

As described in Beauchamp et al. (2021), the aim of the survey was to track a range biological, psychological, and social factors that could affect the mobility and participation of older adults living in the community during the pandemic. Validated and standardized measures, where possible, were used to capture such factors within the multi-measure survey. The International Classification of Functioning, Disability, and Health (ICF), a biopsychosocial model of health that provides a standard language and framework for organizing health-related information (World Health Organization (WHO), 2001), was used to categorize the factors and corresponding measures, in accordance with the objectives of the present study.

*Assessment of Subjective Distress*. The level of distress experienced by older adults living in the community during the first lockdown was measured using the Impact of Events Scale-Revised (IES-R) (Weiss & Marmar, 1997). The IES-R, a valid and reliable measure of subjective distress, consists of 22-items that capture the effects of an event in terms of intrusion (e.g., nightmares; disassociation), avoidance (e.g., numbing; ignoring anything to do with event) and hyperarousal (e.g., anger, irritability). Items are rated using a Likert-scale ranging from *not at all* (0), *slightly* (1), *moderately* (2), *quite a bit* (3), or *extremely* (4). The Cronbach's α for the IES-R scale in this study was 0.92. Scores range between 0 and 88. While the IES-R is not diagnostic for PTSD, scores of ≥ 24 suggest clinical concerns for PTSD, ≥ 33 indicate probable PTSD, and ≥ 37 have been associated with long-term immune suppression (Weiss & Marmar, 1997). For the univariate and multivariate analyses, IES-R scores were dichotomized as “high” if ≥ 24 for clinical interpretability.

*Predictive Factors of Subjective Distress*. Using the ICF, members of the research team (EV, BV) selected and categorized potential variables from the survey based on biopsychosocial factors relevant to distress.

*Contextual Factors.* Personal Factors were age, sex, education, and household income. Education level was dichotomized (*secondary school or less* vs. *some postsecondary or postsecondary*) and household income was categorized into three groups (*less than $20,000*, *$20,000 to $50,000*, and *$50,000 or more*). Environmental factors were loneliness and living arrangements. The frequency of loneliness was assessed using a single item (Irwin et al., 1999) and dichotomized (*< 1 day/week* vs. *at least 1 day/week*) as were living arrangements (*living alone* vs. *living with others*). Using the ICF, resilience has been classified as a Contextual Factor (see Unger et al., 2010) and was measured using the Brief Resilience Scale (BRS; Smith et al., 2008) where scores are categorized as low (*1.00–2.99*), normal (*3.00–4.30*), and high (*4.31–5.00*).

*Functioning and Disability:* Body Functions and Structures were captured and dichotomized using perceived health status (*fair/poor* vs. *good/very good/excellent*), anxiety/depression (*not/slightly* vs. *moderately/severely/extremely*; EQ-5D-5L; Herdman et al., 2011), and nutritional risk (*not at risk* vs. *at high risk*; SCREEN-8 < 38; Keller et al., 2005). To measure Activities, self-reported physical mobility was assessed using the function component of the Late Life Function and Disability Instrument, which is comprised of 32-items that are transformed to a scale from 0 to 100, with higher scores indicating greater function (Haley et al., 2002). For Participation, a global rating of perception of change score (see Beauchamp et al., 2021) for each of the following life areas was categorized into three groups (*a little or much worse* vs. *about the same* vs. *a little or much better*): Ability to do physical activity, keep in touch with others, take care of own health, taking care of errands, and ability to participate in the community and maintain social life.

#### Interview Guide

To invoke reflection of their experiences with daily life after the initial lockdown, the interview guide was structured in accordance with a previous study that examined occupational engagement following a sudden traumatic event, namely a cancer diagnosis (Vrkljan & Polgar, 2001). For the first part of the interview, participants were asked to describe their daily life before the COVID-19 pandemic. The second part referred to their life after the initial lockdown in March 2020 through to the time of their interview. Participants were probed about the occupations they needed to do, wanted to do, and had to do and to describe if and how they participated in such occupations. For the third part of their interview, they were asked to prognosticate what their life might be like after the pandemic and which activities they were most looking forward to doing. Details concerning the development of the interview guide are outlined in Beauchamp et al. (2021) (See Appendix 1 for the full interview guide).

### Recruitment and Procedure

Participants were recruited to participate in the survey using a random digit dialing service from preselected postal codes based on distance from the university. These postal codes were selected based on the municipal census indicating a higher proportion of older adults. Participants had to be aged 65 years and older. They were excluded if they lived in long-term care, or reported having visual, hearing, and/or cognitive impairments that precluded their participation. All survey participants were asked about their willingness to participate in future studies. Of those who responded affirmatively, purposive sampling was used to identify potential interview participants based on the quantitative data with the goal of recruiting individuals that reflected a full range of experienced distress related to the pandemic. A sampling frame was developed and stratified by distress (IES-R; < 24 no concern, ≥ 24 - < 33 clinical concern, ≥ 33 probable PTSD). We sought to interview 8–10 participants from each distress category (Morse, 2000). As we were unsure about the response rate for interviews, particularly those with higher distress, due to the ongoing pandemic, we over-recruited, across distress categories when inviting individuals to be interviewed. Within these strata, participants were also selected based on predictive factors identified from the survey analyses and age, gender, and income to enhance diversity. Those who tested positive for COVID-19 at any point were excluded from the present analysis, as their experience with the public health measures, and/or the health system, would be different from that of other participants. Participants were given the option to participate by telephone or Zoom©. Each interview was conducted by one of two undergraduate members of the research team (ND, RG) and were audio-recorded and transcribed verbatim.

### Data Analysis

For the first objective of the study, we used descriptive statistics for the demographics, IES-R score, and other variables identified as having a potential impact on subjective distress. Multivariable logistic regression was used to identify independent factors associated with high distress measured using dichotomized IES-R score. Candidate factors for inclusion in the regression model were determined based on results from univariate analysis (*p < .2*) and expert judgement. Backward selection was used to establish the final model. Multicollinearity was assessed using variance inflation factor (VIF) where a VIF exceeding 5 indicates high multicollinearity between the independent variable and other variables. Linearity for continuous variables was assessed with respect to the logit of IES-R score via the Box-Tidwell procedure. All analyses used SPSS version 28 and the significance level was set at 0.05.

For the second objective, data analysis was informed by Colaizzi's recommended process for descriptive phenomenology (Morrow et al., 2015). ND and RG reviewed the transcripts to familiarize themselves with the data. Significant quotes of direct relevance to participants’ occupational participation and associated changes, including strategies to manage their engagement, were extracted from the transcripts where the full range of distress scores on the IES-R were considered. Short, concise descriptions (codes) that reflected the meaning attributed to these occupations were identified. Coders engaged in a bracketing process throughout whereby they endeavored to recognize and put aside their presuppositions about the phenomenon as much as possible. Reflexive journaling supported this process after each interview and throughout the analysis. ND and RG met regularly to ensure consensus on coding. Codes were categorized into high-order themes and the transcripts were reviewed again for additional quotes corresponding to these themes. EV, who was not directly involved in data collection or coding, audited the preliminary thematic framework. A narrative summary describing these themes and their structure was drafted by the research team. An older adult, who served in an advisory capacity to the team, reviewed the summary and recommended minor edits to streamline the respective descriptions of the themes (e.g., use bullets rather than paragraph style). The final summary was mailed, or emailed, to participants. BV oversaw data analysis and reviewed quotes, codes, and themes throughout the process. Data were organized and analyzed using qualitative data analysis software (QSR NVivo, version 1). Further details on interview methodology are described using the Consolidated Criteria for Reporting Qualitative studies (see Appendix 2).

## Results

### Survey

For the survey data, 2655 participants were called from the random digit dialing service list. Of these, 312 older adults were recruited (11.75%), 272 completed the baseline survey, with 8 participants (2.94%) excluded due to missing data, and 1 participant (0.37%) who reported being positive for COVID-19. Table 1 details the demographic characteristics of the sample. The majority of participants were women and at least some had post-secondary education. Across the sample, most IES-R and resilience scores were in the ‘normal’ range with the majority reporting none or slight anxiety or depression, as per EQ-5D.

**Table 1.** Demographic Characteristics of Survey Participants and the Sub-Sample Interviewed.

	Survey participants		Interview sample	
Characteristic	*n* (%)	*M* (*SD*)	*n* (%)	*M* (*SD*)
*Sex*				
Female	184 (69.96)		17 (53.13)	
Male	79 (30.04)		15 (46.88)	
*Age, years*		78.04 (7.30)		75.56 (7.62)
65–74	101 (38.40)		17 (53.13)	
75–84	112 (42.59)		13 (40.63)	
85+	50 (19.01)		2 (6.25)	
*Education*				
Less than secondary	30 (11.41)		2 (6.25)	
Secondary	49 (18.63)		4 (12.50)	
Some postsecondary	59 (22.43)		7 (21.88)	
Postsecondary	122 (46.39)		18 (56.25)	
Not available	3 (1.14)		1 (3.13)	
*Household income*				
< $20,000	15 (5.70)		6 (18.75)	
$20,000 to < $50,000	75 (28.52)		7 (21.88)	
$50,000 to < $100,000	70 (26.62)		9 (28.13)	
$100,000+	28 (10.65)		7 (21.88)	
Not available	75 (28.52)		3 (9.38)	
*IES-R score*		13.20 (12.70)		18.22 (16.14)
< 24	218 (82.89)		23 (71.88)	
24 to < 33	21 (7.98)		4 (12.50)	
33 to < 37	8 (3.04)		1 (3.13)	
37+	16 (6.08)		4 (12.50)	
*Anxiety/depression*				
Not or slightly	235 (89.35)		28 (87.50)	
Moderately, severely, or extremely	28 (10.65)		4 (12.50)	
*Resilience*		3.64 (0.74)		3.32 (0.93)
Low	35 (13.31)		10 (31.25)	
Normal	182 (69.20)		14 (43.75)	
High	46 (17.49)		8 (25.00)	

*Note. n* = 263 for survey participants. *n* = 32 for interviewed participants. *M* = mean; *SD =* standard deviation; IES-R = Impact of Event Scale-Revised.

Univariate analysis (see Table 2) identified sex, household income, loneliness, resilience, perceived health, anxiety/depression, nutrition risk, mobility, and perceived changes in ability to keep in touch with others, take care of own health, take care of errands, and participate in the community and maintain a social life, as candidate predictor factors of IES-R (*p* < .2).

**Table 2.** Univariate Analysis Between Factors and Higher Distress as per Scores of ≥ 24 on the IES-R.

			95% CI		*p*
Variable	*n* (%)	OR	*LL*	*UL*	
*Age* ^a^	78.04 (7.30)	1.03	0.98	1.07	.234
*Sex*					
Female	184 (69.96)	ref			
Male	79 (30.04)	0.45	0.20	1.01	.053
*Education*					
Secondary or less	79 (30.38)	ref			
Some post-secondary or post-secondary	181 (69.62)	1.09	0.54	2.21	.810
*Household income*					
< $20,000	15 (7.98)	1.09	0.27	4.38	.904
$20,000 to < $50,000	75 (39.89)	ref			
$50,000+	98 (52.13)	0.55	0.23	1.30	.172
*Loneliness*					
< 1 day/week	172 (65.40)	ref			
1 + day/week	91 (34.60)	3.61	1.86	7.01	<.001
*Live alone*					
Yes	128 (49.42)	ref			
No	131 (50.58)	0.97	0.51	1.86	.933
*Resilience*					
Low	35 (13.31)	9.22	4.14	20.51	<.001
Normal	182 (69.20)	ref			
High	46 (17.49)	0.31	0.07	1.39	.126
*Perceived health*					
Excellent, very good, or good	213 (80.99)	ref			
Fair or poor	50 (19.01)	2.28	1.11	4.72	.026
*Anxiety/depression*					
Not or slightly	235 (89.35)	ref			
Moderately, severely, or extremely	28 (10.65)	7.89	3.42	18.19	<.001
*Nutrition risk*					
Not at risk	97 (36.88)	ref			
At risk	166 (63.12)	2.32	1.09	4.94	.028
*Mobility (LLFDI-FC)* ^b^	61.03 (13.46)	0.96	0.94	0.99	.008
*Perceived changes in ability to engage in*					
*Physical activity*					
A little/much worse	70 (26.72)	1.14	0.56	2.34	.720
About the same	180 (68.70)	ref			
A little/much better	12 (4.58)	0.46	0.06	3.65	.458
*Keep in touch with others*					
A little/much worse	29 (11.07)	1.69	0.63	4.53	.301
About the same	194 (74.05)	ref			
A little/much better	39 (14.89)	2.87	1.30	6.36	.009
*Take care of own health*					
A little/much worse	13 (4.96)	2.40	0.70	8.18	.163
About the same	243 (92.75)	ref			
A little/much better	6 (2.29)	2.70	0.48	15.25	.262
*Take care of errands*					
A little/much worse	73 (27.86)	1.18	0.57	2.42	.660
About the same	180 (68.70)	ref			
A little/much better	9 (3.44)	2.71	0.64	11.49	.175
*Participate in the community and maintain a social life*					
A little/much worse	161 (61.45)	1.15	0.57	2.32	.694
About the same	94 (35.88)	ref			
A little/much better	7 (2.67)	4.29	0.86	21.25	.075

*Note.* OR = odds ratio; CI = confidence interval; *LL* = lower limit; *UL* = upper limit; ref = reference for other categories; LLFDI-FC = Late-Life Function and Disability Instrument-Function Component; IES-R = Impact of Events of Scale-Revised. Covariates with *p *< .2 were included in the full multivariate backward regression model.

^a^ Reflects the mean and standard deviation of age in years as it was measured using a continuous variable.

^b^ Reflects the mean and standard deviation of Mobility (LLFDI-FC) as it was measured using a continuous variable.

The multivariate logistic regression model was statistically significant with high IES-R scores (χ^2^(3) = 46.62, *p *< .001) and explained 27% of the variance in distress scores (see Table 3). Of the 12 predictor variables, only anxiety/depression and resilience were statistically significant. Participants who reported feeling moderately, severely, or extremely anxious (or depressed) had four times the odds of having high distress than those who did not report such concerns. Those with low resilience had almost seven times greater odds of having high distress compared to those with normal resilience.

**Table 3.** Multivariate Logistic Regression Analysis of Factors Associated with a High IES-R ≥ 24.

Variable	OR	95% CI		*p*
		*LL*	*UL*	
*Anxiety/depression*				
Not or slightly	ref			
Moderately, severely, or extremely	4.09	1.60	10.44	.003*
*Resilience*				
Low	6.84	2.95	15.86	<.001**
Normal	ref			
High	0.38	0.08	1.67	.199

*Note.* OR = odds ratio; CI = confidence interval; *LL* = lower limit; *UL* = upper limit; ref = reference for other categories; IES-R = Impact of Events of Scale-Revised. R^2^ (Nagelkerke) = 0.27.

*p < .05. **p < .001.

### Interviews

A total of 32 participants were interviewed between February and August 2021. Interviews ranged between 18 and 115 min (*Mdn* =39.50, *IQR* = 30.75−48.75). Demographic details are outlined in Table 1. Interview participants’ IES-R scores ranged from 0 to 60. Only 9 participants agreed to be interviewed out of our target of 16–20 with IES-R scores indicative of high distress. Since resilience emerged as the strongest predictor of IES-R, the remaining 23 participants) were selected based on a range of BRS scores, as well as gender, age group, and socioeconomic status across the sample (See Table 4).

**Table 4.** Interview Participant Characteristics Predictive of Distress Related to the COVID-19 Pandemic among Interview Participants Scoring High and Normal Distress Scores.

		<24 IES-R (*N* = 23)	≥ 24 IES-R^a^ (*N* = 9)
*Resilience* ^b^		6	4
Low		10	4
Normal		7	1
High			
Anxiety/depression^c^			
None or slight		22	6
Moderate, severe, or extreme		1	3

*Note.* COVID-19 = coronavirus disease 2019; IES-R: Impact of Events of Scale-Revised; PTSD = posttraumatic stress disorder.

^a^ Distress related to the COVID-19 pandemic as measured by the IES-R. Scores where ≥ 24 was categorized as high and suggest clinical concerns for PTSD.

^b^ Brief Resilience Scale; low =1.00–2.99, normal = 3.00–4.30, and high = 4.31–5.00.

^c^ Anxiety or depression measured by item 5 on the EQ-5D-5L.

**Figure 1.**
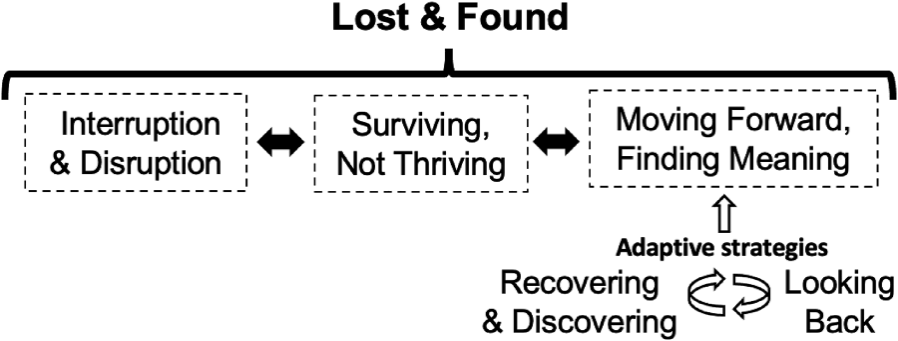
Conceptualization of the main theme and subthemes from interviews with community-dwelling older adults concerning their occupational participation during the coronavirus disease 2019 (COVID-19) pandemic. *Note.* As described in the results, some participants did not move across this process. As such, the dotted lines denote the permeability of these experiences, as do the bidirectional arrows. To move forward, the participants enacted adaptive strategies in different ways, which are depicted using the white arrows.

From the qualitative analysis, the overarching theme, “Lost & Found” reflected how the participants navigated changes in their occupational participation during the pandemic. At the time of the first lockdown, participants experienced an immediate and profound ‘interruption and disruption’ to their occupational participation. With the loss of many meaningful occupations, participants described “surviving, not thriving” during this period. Some drew upon adaptive strategies, “recovering and discovering” and “looking back,” as they moved forward, or tried to move forward, to engage in meaningful occupations. However, not all participants were able to identify and engage in such occupations. Some participants remained stuck in an existence of survival. They described how they waited for the pandemic to pass. Figure 1 illustrates the main theme in relation to the subthemes that emerged from this analysis, which are explicated below using selected quotes. Within the quotes, IES-R and BRS scores, alongside age and gender of the participants, are provided.

#### Lost and Found

Across participants, the overarching theme of ‘Lost and Found’ described how the sudden ‘loss’ in occupational routines was managed (i.e., interruption and disruption). Each participant had a story of not being or feeling meaningfully occupied at the outset of the pandemic and how this impacted their wellbeing (surviving, not thriving) and of trying to “find” meaningful occupations in which they could participate safely (moving forward, finding meaning). As described in the subtheme “moving forward, finding meaning,” some participants shared how they enacted adaptive strategies (i.e., Recovering and Discovering, Looking Back) to manage their occupational participation, whereas others struggled to adapt their occupational pursuits and find meaningful occupations in response to their changed circumstances. These strategies and differences in the experiences of participants are further described under this subtheme.

*Interruption and Disruption.* At the outset of the pandemic, each of the participants recalled how their occupational participation changed in an instant, as one participant indicated, “*…water aerobics, bridge… lunches, coffee hour… all gone”* (P6: Male, age 84, IES-R 6, normal BRS 4). Another described how usual routines were suddenly disrupted, “*…* Sometimes we wake up in the morning and we will look at each other and say, ‘what the hell are we going to do today?’” (O151: male, age 70, IES-R 19, low BRS 2.17). Some participants had difficulty coping with this disruption, as one shared, “*…* no motivation to get up because there is not much to do… we cannot do anything because we are supposed to stay home.” (P12: Female, age 76, IES-R 24, normal BRS 3.17). Their respective descriptions contrasted with daily life before the pandemic where words like “busy,” “active”, and “full” were used. However, for a few participants, life under quarantine did not change much from life before the pandemic, as one participant related, “As far as meeting other people, I was not interested in that for some years ..*.*” (O73: male, age 95, IES-R 10, normal BRS 3.17).

*Surviving, not Thriving.* As days in lockdown turned from days into weeks, the focus was on just trying to get through it, as one participant expressed, “I am tolerating it rather than enjoying it.” *(*P103: Male, age 72, IES-R 11, normal BRS 3.5*).* “Quiet” was commonly used to describe this time alongside variations of the word, “boredom” where the struggle with monotony was shared across participants with a range of distress scores: “… very routine and very dull” (O159: Male, age 83, IES-R 7, low BRS, 2.83) and “… boring and monotonous …” (O25, Male, age 79, IES-R 26; normal BRS: 3.67). In these early days, participants indicated they felt “restricted” in their occupational participation from which some emphasized the importance of persevering, as one participant articulated, “.*..* restricted that is a good word for it, restricted, but not giving up…it has reduced the activity, but not quite cancelled it.” (O151: Male, age 70, IES-R 19, high BRS 0), whereas another participant shared how she didn’t see a way forward at that time. She equated these restrictions with incarceration, “… it becomes like a prison because you do not leave your apartment … you become suspicious of everything and everybody, which is bad …” (O8: Female, age 74, IES-R 59, normal BRS 3.17).

*Moving Forward, Finding Meaning.* When participants realized, the lockdown was going to go on longer than first expected, they tried to find ways to participate in their respective occupations. Two main adaptive strategies emerged: (1) Recovering and Discovering and (2) Looking Back.

*Recovering and Discovering.* Finding new ways to participate in previous occupations was necessary due to public health measures that restricted many out-of-home activities. Some recovered such occupations by shifting to virtual environments, as one participant described, “*…* one granddaughter… she’ll just FaceTime me from her laptop, and I count on that… she just brightens up my whole day.” (O24: Female, age 76, IES-R 8, normal BRS 3). Others detailed how they adapted their favorite activities during lockdown, such as going out for a meal, “… we would get take-out from different restaurants … I would set up the dining room table with my good dishes, candles, and music in the background to pretend we were in an actual restaurant.” (O130: Female, age 82, IES-R 15, normal BRS 3.83)*.* Some discovered new ways to find meaning and feel connected to their community but doing so safely. For example, a participant (O151: Male, age 70, IES-R 19, High BRS 0) shared what he coined, “policeman coffees*.*” He described how he and his son purchased their respective beverages, parked their cars six feet apart, and opened their windows to chat. While adaptations and new occupations led to recovering meaningful engagement for some, for others the adaptations or new experiences were perceived less positively, as one participant shared that socializing now meant, “… relying on the computer, which I hate.” (P12, female, age 76, IES-R 24, normal BRS 3.17). Another participant learned to play a new instrument during the lockdown, yet when was asked about changes she made to activities during the pandemic, she replied flatly “nothing new” (O8, Female, age 74, IES-R 59, Normal BRS 3.17).

*Looking Back.* When reflecting on what helped them get through this challenging time, participants shared how they found themselves recalling recent and past adversities as a reminder that they could get through it. For example, one participant described how his job had been tough at times from which he drew strength, “I am used to being in isolation. I learned how to cope pretty good that way …” (P115: Male, age 66, IES-R 22, Normal BRS 3). A few participants described how navigating the recent loss or illness of a loved one was a reminder that life does indeed go on, “… my youngest … passed away 2 years ago … that was difficult … but life goes on and you need to deal with it” (O109: Female, age 82, IES-R 20, High BRS 0). Another shared her determination with moving forward despite personal hardships she had recently experienced:

I lost my husband at the beginning of this. I could have gone one or two ways, either you go into a deep depression over the loss, or I could say … I do not have to worry about him anymore, I can put my energies into other things in my life and COVID is not going to stop me from that. (O41: Female, age 79, IES-R 0, low BRS 1).

Although some participants shared how they found themselves looking back to find strength from past adversities, they compared their current slate of occupations to that of their past. A participant remarked how the opportunity to connect socially through such occupations was missing, “… the normal things we did … I miss all that … I miss my friends*.*” (P105: Female, age 81, IES-R 60, low BRS 2.67). Another expressed that the time they had lost due to the pandemic was time they were never going to get back, which was difficult to reconcile,

It [the pandemic] is worse for older people because when you are young. If you miss something you still have 25–30 years to do that but when you are older you do not have 25–30 years to do something you missed in those years. It is just not going to happen again. So, it is almost like you lost part of your life.

(O8: Female, age 74, IES-R 59, normal BRS 3.17)

For this participant and a few others, the focus was more on what was missing in terms of their past occupations, rather than drawing strength from such adversity. As a result, some remained stuck, unable to move forward, as they waited for the lockdown to be lifted.

## Discussion

The results demonstrated that low resilience was most predictive of high distress alongside anxiety and depression, as reported by older adults living in the community during the first lockdown of the COVID-19 pandemic. The present study found 17% of survey respondents experienced distress at a level that met the criteria for PTSD, which is almost four times higher than the estimated lifetime prevalence for community-dwelling older adults reported prior to the pandemic (Cook et al., 2017). From our interviews, the main theme of ‘Lost and Found’ and corresponding sub-themes described how the participants, including those with high distress, navigated the sudden interruption and disruption in their occupational participation during the first lockdown. However, some described difficulty enacting adaptive strategies that helped others move forward. For these participants, they focused on what was missing from their current circumstances. As a result, they were not moving forward or finding meaning per se and remained in a place of “survival” and wishing the pandemic was over when they envisioned a resumption in meaningful occupations they had done when life was “normal.” Hence, the current study is unique in not only identifying factors associated with high distress but also how occupational participation was managed during this challenging time, as described by those who reflected a range in level of distress.

Our survey analysis identified lower resilience as a risk factor for high distress. While few studies have examined the relationship between resilience and distress related to the COVID-19 pandemic beyond first responders and healthcare workers, a recent meta-analysis of the relationship between resilience and a broader conceptualization of distress (including stress, anxiety, and depression) found a negative robust association between resilience and distress (Jeamjitvibool et al., 2022). This relationship was consistent across sub-populations, including the general population, and patients, and is congruent with our findings among older adults. Resilience has been defined as “the capacity of individuals to navigate the psychological, social, cultural and physical resources that sustain their well-being” (Unger, 2006, p. 225). Using the BRS scale, which measures of one's ability to “bounce back” during times of major distress (Smith et al., 2008), our findings suggest that those with lower resilience could be more susceptible to adverse outcomes, including high distress. Although feelings of distress should be expected during times of major upheaval, such as the pandemic, the concern is when one's level of stress moves beyond what might be considered typical under the circumstances. For older adults with low or no resilience, like some of the participants in the current study, the long-term consequences of prolonged distress on emotional well-being, including PTSD, could be particularly concerning (Cook et al., 2017).

The current study also found that the severity of anxiety or depression was independently associated with distress related to the COVID-19 pandemic. It should be noted that the level of anxiety or depression in the present study was not different from prevalence observed in the population prior to the pandemic. Canadian research using the EQ-5D-5L have reported similar distributions related to the anxiety/depression item prior to the COVID-19 pandemic in studies of the general population where 79.2%−86.2% reported none or slight anxiety or depression and 13.6%−20.9% reported moderate, severe, or extreme anxiety or depression (Alberta PROMs & EQ-5D Research & Support Unit, 2018; Poder et al., 2020).

The present study complements the work by Rotenberg et al. (2021) in Canada and Calrsson et al. (2022) in Sweden by enhancing our understanding of how community-dwelling older adults managed daily life during the first lockdown. More specifically, our methodology enabled the inclusion of participants who were experiencing diverse levels of distress related to the pandemic with some experiencing extremely high distress. This sampling approach provided unique insights into how older adults with a range of distress managed occupational participation that has not been considered in previous qualitative studies of their daily life under lockdown. Some participants described how they invoked adaptive strategies where many were able to recover past occupations or discover new ones that were meaningful despite the challenging circumstances. Both Rotenberg et al. and Carlsson et al. also described how participants in their studies used various strategies, albeit in slightly different ways. For example, Rotenberg et al. identified how individuals engaged in certain occupations that helped maintain a more positive affect, such as listening to music, eating a favorite food, reading, and playing games. Interestingly, the authors concluded that these new daily activities tended to be more sedentary and less meaningful. Conversely, Carlsson et al. identified that participants in their study engaged in meaningful occupations during the first 100 days of the pandemic. Using the dimensions of occupation (i.e., doing, being, becoming, belonging), Carlsson et al. noted such occupations were wide-ranging from more active pursuits, such as gardening, to sedentary activities, such as reading and talking to others by phone or web conference. Although these same dimensions were not used in the present study, the participants similarly described engaging in an array of occupations that were valued during this challenging time.

The present study found that all participants used strategies to engage in occupations during the first lockdown, but not all were successful in finding *meaningful* occupation. In her seminal article that discusses the dimensions of meaning in the occupations of daily life, Hammell (2004) emphasizes that meaning can be derived from the feelings experienced by the individual during the occupational engagement, as opposed to focusing on the outcome or purpose of such engagement. Using examples of what she referred to as “life-change events” alongside research evidence, Hammel highlighted the critical role of occupation at times of major disruption where “… both the consequences and significance of the disruption might be changed through occupation” (p. 302). In turn, she implored occupational therapists and others to use these dimensions to identify occupations and inform interventions during such events, rather than using predetermined categories that focus on outcome and purpose, such as productivity, self-care, and leisure. However, the process by which one comes to ascribe value to certain occupations, and not others, is highly complex and individualized. Hence, drawing on the dimensions of occupation to identify those that are valued by the person in question and, in turn, facilitating engagement where possible and appropriate, could help manage changes in times of major disruption, such as another pandemic.

There were participants in the current study who recovered or discovered new occupations, such as playing an instrument, yet did not seem to ascribe meaning to this experience. While level of distress and/or external forces, such as the implementation of public health measures, may have circumvented the ability for participants to find occupations or express meaning in the same way as before the pandemic, the lack of stimulation in the environment could be akin to occupational deprivation. Scholars in occupational science have framed such deprivation as not only an occupational injustice but also occupational displacement where forces external to the individual can prevent meaningful occupational participation with implications on well-being (see Whiteford et al., 2020). For those who described difficulty adapting to their changed circumstances and remained stuck in an existence of “surviving, not thriving,” there is a concern for their psychological well-being. However, further research is necessary to confirm a relationship between occupational disruption, distress, and well-being.

### Study Strengths and Limitations

The mixed methods approach undertaken in the present study had many strengths. For example, results from the standardized measures used in the survey analyses not only identified those factors associated with high distress, but also informed the sample selected for the qualitative interviews by identifying informants based on their quantitative data (i.e., IES-R, BRS, age, gender). During a major event, such as a pandemic, there is the possibility that the individual experience of psychological distress can be minimized, meaning those with high distress may assume that everyone is experiencing a similar level of duress. Using a valid measure of psychological distress enabled our team to carefully explore participants’ occupational participation in light of their IES-R scores and the associated implications.

Caution is warranted regarding the interpretation of findings. We were unable to recruit our target sample size for the interviews regarding high distress. Although those who responded to the survey reflected a range in terms of age, gender, education, and other variables, they were predominantly of higher educational status, and, as such, may not be representative of the experiences of older Canadians living in the community during this time. Unfortunately, we neglected to ask participant ethnicity, and some also did not want to share income information. The baseline survey data were collected across 4 months, meaning the level of subjective distress reported by those closer to the outset of the pandemic may have differed from those who responded later in the same period (Beauchamp et al., 2021). A single interview was conducted with each participant 6–12 months after the first lockdown when data were collected from the baseline survey. While recall bias is an important consideration, utilizing a retrospective approach may have provided time to reflect on how they navigated their occupational participation during that challenging period.

## Conclusion

Lower resilience and anxiety/depression are most strongly linked to high distress among community-dwelling older Canadians surveyed during the first lockdown of the COVID-19 pandemic. Themes from the interviews with those who ranged in level of distress indicated that the lockdown and associated public health measures disrupted daily life where some participants enacted strategies that helped them move forward and find meaning, whereas others experienced ongoing challenges. A sub-group analysis of those who experienced such challenges may help to identify resources that support health and well-being during major life change events. Although many in the current study were able to manage changes in their occupational participation by invoking such strategies, some remained “stuck,” unable to move forward, as they waited and hoped for the pandemic to pass.

This study is unique in that it included the voice of those with high distress levels who are often excluded from such research. A highly individualized approach may be needed to address occupational participation in times of major disruption. Given the emergence of new variants and the potential of a future pandemic and/or in the event of another large-scale natural or human-made disaster, findings indicate that some older adults may be at a higher risk for distress during such disruptions if they have no or low resilience and/or are experiencing anxiety/depression. As such, occupational therapists and others can play an important role with those more vulnerable to distress and other adverse outcomes. By facilitating engagement in occupations identified as critical and meaningful, they can support the well-being of individuals and do so in ways that are both safe and appropriate under the circumstances in question.

### Key Messages

- Lower resilience and anxiety/depression were most predictive of high distress among community-dwelling older Canadians surveyed during the first COVID-19 lockdown.
- While many older adults, including those with high distress, managed changes in their occupational participation, some experienced ongoing challenges adapting to daily life under lockdown.
- With the potential for new variants or another large-scale disruption in the future, further understanding of such challenges to identify resources that can support occupational participation and well-being is needed.

## Supplemental Material

sj-docx-1-cjo-10.1177_00084174231165832 - Supplemental material for Exploring Distress and Occupational Participation Among Older Canadians During the COVID-19 Pandemic Supplemental material, sj-docx-1-cjo-10.1177_00084174231165832 for Exploring Distress and Occupational Participation Among Older Canadians During the COVID-19 Pandemic by Elisabeth Vesnaver, Nicholas Dietrich, Renata Kirkwood, Jinhui Ma, Rhianna Guennel, Marla Beauchamp, Heather Keller, Luciana Macedo, Janie Astephan Wilson and Brenda Vrkljan in Canadian Journal of Occupational Therapy

sj-docx-2-cjo-10.1177_00084174231165832 - Supplemental material for Exploring Distress and Occupational Participation Among Older Canadians During the COVID-19 Pandemic Supplemental material, sj-docx-2-cjo-10.1177_00084174231165832 for Exploring Distress and Occupational Participation Among Older Canadians During the COVID-19 Pandemic by Elisabeth Vesnaver, Nicholas Dietrich, Renata Kirkwood, Jinhui Ma, Rhianna Guennel, Marla Beauchamp, Heather Keller, Luciana Macedo, Janie Astephan Wilson and Brenda Vrkljan in Canadian Journal of Occupational Therapy
