# Supplementary material for: Exploring Distress and Occupational Participation Among Older Canadians During the COVID-19 Pandemic
Source: Can J Occup Ther. 2023 May 15;90(2):173–84. doi: 10.1177/00084174231165832 (PMC10189528; doi:10.1177/00084174231165832)
Supplement: sj-docx-1-cjo-10.1177_00084174231165832 - Supplemental material for Exploring Distress and Occupational Participation Among Older Canadians During the COVID-19 Pandemic [file sj-docx-1-cjo-10.1177_00084174231165832.docx]

# Appendix 1 – Semi-structured interview guide

Thanks again for agreeing to be part of our survey and these follow-up interviews. As mentioned on the phone, we are interested in hearing about your life prior to and during the pandemic as well as ask you questions about what you think your life will be like after the pandemic is over. To help guide the interview, we want you to think about your life as though we were making a movie trilogy where the first part of your trilogy is your life ‘before COVID-19.’ The second part of the trilogy refers to your life after the initial the lockdown started in March 2020 to your current life as it is now. For the third and final part, we will ask you to look ahead to what you think your life might be like in the post-pandemic period.

Does this still sound okay to you? Do you have any questions before we start?

Also, as we’d discussed, in order to help with the analysis, I am going to record our discussion. All identifiable information will be removed from the data.

Are we ready? I am going to start the recording now.

(Please note the bulleted italicized questions signify additional probes used, when appropriate)

**Part 1 of Trilogy: Reflecting on your life before the COVID-19 pandemic**

**For this first part of this interview, we are going to ask you reflect on your life before the COVID-19 Pandemic.**

1. Can you describe a typical day for you prior to the pandemic and associated public health measures were in place?
2. Can you tell me a little bit about your social activities before the pandemic?
   - *Social activities usually refer to those activities that you do for fun and involve others. What were the types of activities in which you participated before the pandemic?*
3. Who would you describe as having an important role in your day-to-day life before the pandemic?
   - *People who might be important in your life could be family members, such as spouse, or children or siblings, or could be friends. Can you share with me anyone who you considered to be important in your life before the pandemic?*
   - *Why were these individuals important to you before the pandemic?*
4. In one or two words, how would you describe your life before the pandemic? Can you elaborate on why you chose those particular words?
   - *If you are having trouble thinking of words, can you describe in 1-2 sentences about how felt about your life before the pandemic?*

**Part 2: Life During the COVID-19 Pandemic**

**For this next part of this interview, we are going to ask you questions about your experiences since the pandemic began in March 2020 until now.**

1. Reflecting on your life during the COVID-19 pandemic, can you describe what a typical day is like for you now?

- *What has changed (or not) about your life since the COVID-19 restrictions were put in place?*
- *How have you adapted (or not) your everyday activities since the pandemic began?*
- *Can you share any strategies/resources that you have found particularly helpful with managing your activities during the pandemic?*

1. Since the pandemic began, can you tell me about your social activities and network?
   - *Who would you describe as having an important role in your life since the pandemic began? Can you tell me more about why they are important to you?*

1. In one or two words, how you describe your life during the pandemic? Can you elaborate on why you chose these words?
   - *If you are having trouble thinking of words, can you describe in 1-2 sentences about how feel about your life during the pandemic?*
2. All things considering, how do you think you have handled the restrictions imposed by the pandemic?
   - *If you had to pick a rating on a scale of 1-10 where 1 refers to not handling the pandemic well at all, and 10 refers to exceptional handling of the pandemic, how would you rate yourself using this scale? Can you elaborate on why you rated yourself in this way?*
   - *Can you elaborate on why you feel you have handled the pandemic well? (or not well, depending on answer)?*
3. If someone in a high level of government asked you about how you are coping during the pandemic, what might you say drawing on your own personal experiences during the pandemic?
   - *We are interested in your own views, not what you have heard from friends or what you have read in the news, but your own experiences since the pandemic began.*
   - *Based on your experience, is there anything that you might suggest that could help you during pandemic?*

**Part 3: Looking ahead to Life post-COVID-19**

**For this final part of the interview, we are going to ask you questions that ask you to consider your life might be like in the post-pandemic period.**

1. Looking ahead, what do you think your life will look like once the pandemic is over?
   - If answer indicative that ‘life will stay the same as it is now (i.e., during pandemic)’ – Follow-up: *Why do you think your life won’t change from how it is now?*
   - If answer indicative that of return to pre-pandemic life – Follow-up: *Why do you think your life will be the same as it was before?*
   - If answer indicative ‘being different to what life was previously?’ – Follow-up: *Why do you think your life will be so different?*
2. In one or two words, how might you describe your life after the pandemic is over? Can you elaborate on why you chose these words?
   - *If you are having trouble thinking of words, can you describe in 1-2 sentences about how you feel your life will change after the pandemic is over?*

1. What activity, if anything, are you most looking forward to doing once the pandemic is over? Why are you looking forward to this particular activity?
   - *If you are having a hard time thinking of an activity is there an activity you used to do that you hope you will be able to do again?*

**Closing questions:**

Is there anything else that you would like to share that you didn’t get a chance to do so in the above questions.

Would it be ok if we followed up with you about some of your responses if we require more information? [yes or no]

**Thank you again for doing this interview.**
