# Supplementary material for: Exploring Distress and Occupational Participation Among Older Canadians During the COVID-19 Pandemic
Source: Can J Occup Ther. 2023 May 15;90(2):173–84. doi: 10.1177/00084174231165832 (PMC10189528; doi:10.1177/00084174231165832)
Supplement: sj-docx-2-cjo-10.1177_00084174231165832 - Supplemental material for Exploring Distress and Occupational Participation Among Older Canadians During the COVID-19 Pandemic [file sj-docx-2-cjo-10.1177_00084174231165832.docx]

Appendix 2

**Consolidated criteria for reporting qualitative studies (COREQ): 32-item checklist**

**Manuscript:** Exploring Distress and Occupational Participation among Older Canadians during the COVID-19 Pandemic

Developed from:

Tong A, Sainsbury P, Craig J. Consolidated criteria for reporting qualitative research (COREQ): a 32-item checklist for interviews and focus groups. International Journal for Quality in Health Care. 2007. Volume 19, Number 6: pp. 349 – 357

| **No. Item** | **Guide questions/description** | **Study Details** | **Reported on Page #** |
| --- | --- | --- | --- |
| **Domain 1: Research team and reﬂexivity** |  |  | P# to be updated with proofs |
| *Personal Characteristics* |  |  |  |
| 1. Inter viewer/facilitator | Which author/s conducted the inter view or focus group? | ND and RG | p8 |
| 2. Credentials | What were the researcher’s credentials? E.g. PhD, MD | Interviewers were undergraduate researchers at the time of data collection. | P8 |
| 3. Occupation | What was their occupation at the time of the study? | Undergraduate students | P8 |
| 4. Gender | Was the researcher male or female? | One identified as male and one as female | - |
| 5. Experience and training | What experience or training did the researcher have? | The interviewers each had taken an undergraduate research methods course which included a module on qualitative methods. Both received focused training from the senior author (BV) in interviewing skills and data analysis for this project. (BV) is a Professor with experience in phenomenology. The first author (EV), is a postdoctoral fellow at the, with training in qualitative methodology and experience with grounded theory methodology and reflexive thematic analysis. EV also advised on the interview and analysis process and audited the final thematic framework. As a key resource, an interviewing manual was created for this study detailing key information required to undertake this study. Interviews were each given a copy of the manual.  Interviewer training is detailed in the published protocol. | - |
| *Relationship with participants* |  |  |  |
| 6. Relationship established | Was a relationship established prior to study commencement? | All interview participants had participated in the quantitative survey and had contact with members of the wider research team. Three people had been interviewed for the survey by ND, but there was no notable difference in the interview. It is possible that the previous interaction facilitated recruitment. | - |
| 7. Participant knowledge of the interviewer | What did the participants know about the researcher? e.g. personal goals, reasons for doing the research | The interviewer introduced themselves as a research assistant on the larger study entitled “Impact of COVID-19 and social distancing on the mobility and participation of older adults living in Hamilton, Ontario.” | - |
| 8. Interviewer characteristics | What characteristics were reported about the inter viewer/facilitator? e.g. Bias, assumptions, reasons and interests in the research topic | Participants were explained the aims of the interview study. Specifically, they were told “This study aims to explore the lived experiences of community-dwelling older adults like yourself, before, during, and after the pandemic to gather insights on factors that contribute to your ability to manage your daily life during the pandemic.” No frameworks or opinions about the research topic were shared by the interviewer. | - |
| **Domain 2: study design** |  |  |  |
| *Theoretical framework* |  |  |  |
| 9. Methodological orientation and Theory | What methodological orientation was stated to underpin the study? e.g. grounded theory, discourse analysis, ethnography, phenomenology, content analysis | Colaizzi’s Descriptive Phenomenology guided the qualitative component of the study. | P4 |
| *Participant selection* |  |  |  |
| 10. Sampling | How were participants selected? e.g. purposive, convenience, consecutive, snowball | Purposive sampling was used to identify potential interview participants based on the quantitative data with the goal of recruiting individuals that reflected a full range of experienced distress related to the pandemic. A sampling frame was developed and stratified by distress (IES-R; <24 no concern, ≥24- <33 clinical concern, ≥33 probable PTSD). We sought to interview 8-10 participants from each distress category (Morse, 2000). Within these strata, participants were also selected based on predictive factors identified from the survey analyses and age, gender, and income to enhance diversity. | P13 |
| 11. Method of approach | How were participants approached? e.g. face-to-face, telephone, mail, email | Participants were phoned. | P5 |
| 12. Sample size | How many participants were in the study? | Thirty-two participants were in the interview study. | P11 |
| 13. Non-participation | How many people refused to participate or dropped out? Reasons? | 54 survey participants were invited to participate in the interview study; 22 declined or could not be reached; 32 consented and participated in the study. | - |
| *Setting* |  |  |  |
| 14. Setting of data collection | Where was the data collected? e.g. home, clinic, workplace | Research assistants interviewed participants by phone. Interviewers conducted interviews from their home offices (in private areas). Phone calls were made using Avaya client through the institution and with an institution number. | P8 |
| 15. Presence of non-participants | Was anyone else present besides the participants and researchers? | Researchers were alone in a private workspace. It was not collected whether participants had others in their home at the time of the interview. | - |
| 16. Description of sample | What are the important characteristics of the sample? e.g. demographic data, date | Sex, age, education, household income, psychological distress score, current anxiety or depression, score on Brief Resilience scale were reported. | Table 1 |
| *Data collection* |  |  |  |
| 17. Interview guide | Were questions, prompts, guides provided by the authors? Was it pilot tested? | The question guide is appended as a supplemental file. | Supplemental File 1 |
| 18. Repeat interviews | Were repeat inter views carried out? If yes, how many? | A single interview with each participant was conducted. | P8 |
| 19. Audio/visual recording | Did the research use audio or visual recording to collect the data? | Interviews were audio-recorded. | P8 |
| 20. Field notes | Were ﬁeld notes made during and/or after the inter view or focus group? | Field notes were collected after the completion of each interview to capture initial thoughts and views. Reflexive journaling was used to consider how analysts’ lived experiences shaped the interpretation and supported the bracketing process of the analysis. | P9 |
| 21. Duration | What was the duration of the inter views or focus group? | Interviews ranged between 18 and 115 minutes (Mdn = 39.50, IQR =30.75 – 48.75) | P11 |
| 22. Data saturation | Was data saturation discussed? | No | - |
| 23. Transcripts returned | Were transcripts returned to participants for comment and/or correction? | A summary of the findings was sent to participants. | P10 |
| **Domain 3: analysis and ﬁndings** |  |  |  |
| *Data analysis* |  |  |  |
| 24. Number of data coders | How many data coders coded the data? | Two data coders coded the data. |  |
| 25. Description of the coding tree | Did authors provide a description of the coding tree? | The higher order themes are identified in Figure 1, with lower order themes identified in the results section. | Figure 1  P10-16 |
| 26. Derivation of themes | Were themes identiﬁed in advance or derived from the data? | Themes were inductively identified from the data. |  |
| 27. Software | What software, if applicable, was used to manage the data? | Data analysis was conducted using NVivo, version 1. | P14 |
| 28. Participant checking | Did participants provide feedback on the ﬁndings? | No | - |
| *Reporting* |  |  |  |
| 29. Quotations presented | Were participant quotations presented to illustrate the themes/ﬁndings? Was each quotation identiﬁed? e.g. participant number | Participants were identified with participant id. Key demographic data including sex, age, psychological distress score, and resilience score was provided with quotes. | P10-16 |
| 30. Data and ﬁndings consistent | Was there consistency between the data presented and the ﬁndings? | Yes | P10-16 |
| 31. Clarity of major themes | Were major themes clearly presented in the ﬁndings? | Yes, major themes are presented in text and in Figure 1. | P10-16 |
| 32. Clarity of minor themes | Is there a description of diverse cases or discussion of minor themes? | Yes, minor themes are discussed within each major theme. | P10-16 |
